# Hypercalcemia and acute kidney injury induced by eldecalcitol in patients with osteoporosis: a case series of 32 patients at a single facility

**DOI:** 10.1080/0886022X.2019.1578667

**Published:** 2019-03-26

**Authors:** Seishi Aihara, Shunsuke Yamada, Hideaki Oka, Taro Kamimura, Toshiaki Nakano, Kazuhiko Tsuruya, Atsumi Harada

**Affiliations:** a Division of Kidney Center, Matsuyama Red Cross Hospital, Matsuyama, Japan;; b Department of Medicine and Clinical Science, Graduate School of Medical Sciences, Kyushu University, Fukuoka, Japan;; c Department of Nephrology, Nara Medical University, Nara, Japan

**Keywords:** Acute kidney injury, eldecalcitol, hypercalcemia, osteoporosis

## Abstract

**Background:** Eldecalcitol (ELD) is an active vitamin D_3_ analog that is widely used in Japan for the treatment of osteoporosis. The most common adverse drug reaction of ELD is hypercalcemia. However, few reports have focused on acute kidney injury (AKI) associated with ELD-induced hypercalcemia.

**Materials and methods:** We retrospectively reviewed the medical records at our hospital for cases of hypercalcemia-induced AKI between April 2013 and February 2018. Among them, we focused on patients who developed AKI secondary to ELD-induced hypercalcemia.

**Results:** Among 69 patients who developed hypercalcemia-induced AKI, 32 patients (46.4%) developed AKI associated with ELD-induced hypercalcemia. Their mean age was 82 ± 5 years, 97% of them were female, mean corrected serum calcium level was 12.2 ± 1.5 mg/dL, serum creatinine level was 2.5 ± 2.2 mg/dL, and estimated glomerular filtration rate was 23.9 ± 14.4 ml/min/1.73 m^2^ on admission. ELD administration was discontinued in all patients and some of them were treated with hydration with or without calcitonin, which was followed by a normalization of serum calcium level. Corrected serum calcium level on admission was significantly higher (*p* < .05) in patients treated with magnesium oxide. Although there were no significant differences, serum calcium and creatine levels on admission tended to be higher in patients who were treated with other drugs that affect renal hemodynamics and renal calcium metabolism than those not taking these drugs.

**Conclusions:** Prescribers of ELD should regularly monitor serum calcium levels and kidney function to prevent hypercalcemia and AKI associated with ELD and pay more attention to concomitant drugs especially magnesium oxide.

## Introduction

Osteoporosis is a common disease characterized by decreased bone strength, low bone mass, and skeletal fragility, which increase the risk of bone fracture [1]. In Japan, older subjects make up 26.7% of the total population, and osteoporosis in the older generation is a major public health problem [2]. To date, there have been several classes of drugs prescribed for patients with osteoporosis. Among them, bisphosphonates, which inhibit bone resorption and maintain a positive bone-remodeling balance [3], have been used as a first-line treatment for osteoporosis because they have been shown to greatly reduce the risk for bone fracture. However, bisphosphonates have the potential to cause serious side effects such as osteonecrosis of the jaw, atypical femoral fracture, and esophageal ulcer, and are not always the best treatment option. Hence, some of the patients with osteoporosis are still being treated with vitamin D receptor activators (VDRAs).

Eldecalcitol (ELD), a type of VDRAs widely used in Japan for the treatment of osteoporosis, was demonstrated to be superior to alfacalcidol (ALF), another VDRA often used for the treatment of osteoporosis [4–6]. A 3-year, randomized, double-blind clinical trial showed that compared with ALF treatment, ELD significantly decreased the incidence of vertebral fractures and wrist fractures, and led to greater increases in lumbar spine and total hip bone mineral density and a stronger suppression of bone turnover markers [5]. Another clinical study showed that ELD has an advantage over ALF in the improvement in bone geometry and biomechanical parameters in osteoporotic patients [6]. Like other vitamin D analogs, one of the important adverse events associated with ELD is hypercalcemia [7]. A recent survey conducted in Japan reported on the real-world prevalence of ELD-induced hypercalcemia (111/528 cases, 21%, and 29/3285 cases, 0.88%) [5–7]. However, based on our personal experience, patients treated with ELD develop hypercalcemia more frequently than has been reported, especially female patients with chronic kidney disease (CKD), those with lower body weight, and those treated with drugs such as non-steroidal anti-inflammatory drugs (NSAIDs), renin-angiotensin-aldosterone system inhibitors (RAASIs), or diuretics. Furthermore, the incidence of ELD-induced acute kidney injury (AKI) has not been well investigated. The purpose of our study was to examine the clinical course and characteristics of patients with hypercalcemia and AKI induced by ELD at the time of admission at a single facility.

## Materials and methods

### Study design and participants

We retrospectively reviewed medical records to identify patients who developed hypercalcemia-induced AKI at our hospital between April 2013 and February 2018. Among patients with hypercalcemia-induced AKI, we focused on the cases of AKI associated with ELD-induced hypercalcemia. Initially, all of the patients included in the present study were outpatients referred to our facility by clinicians working at other clinics and hospitals, and then they were hospitalized after consultation for the treatment of hypercalcemia and resulting AKI. The study protocol was approved by the local ethics committee of Matsuyama Red Cross Hospital (No.688) and was registered at the clinical trial registry (UMIN. R000033630). The study was performed according to the Ethics of Clinical Research (Declaration of Helsinki). Informed consent was obtained from each patient prior to study participation.

To determine the impact of hypercalcemia on serum creatinine (Cr) change, we examined the correlation between Δ serum Cr and serum calcium (Ca) level on admission and Δ serum Ca level, where Δ serum Cr and Δ serum Ca were calculated by the following formula; Δ serum Cr (mg/dL) = (serum Cr level on admission) – (the lowest serum Cr level during hospitalization), Δ serum Ca (mg/dL) = (serum Ca level on admission) – (the lowest serum Ca level during hospitalization).

### Definitions of AKI, hypercalcemia, and treatments

AKI was defined as an increase in serum Cr level from the baseline serum Cr level, according to the AKI criteria defined by Kidney Disease Improving Global Outcomes (KDIGO) guidelines of 2012: increase in serum Cr by ≥0.3 mg/dL within 48 h, an increase in serum Cr to ≥1.5 times baseline within 7 days, or urine volume <0.5 mL/kg/hour for 6 h [8]. When the serum Cr level at baseline was unknown, it was assumed to be the lowest serum Cr level during the hospitalization [9]. Outpatient serum Cr levels were used as the baseline serum Cr levels in 30 patients and the lowest serum Cr levels during the hospitalization were used as the baseline serum Cr levels in 2 patients, as outpatient serum Cr levels were missing in those 2 patients. Hypercalcemia was defined in the current study as corrected Ca concentration >10.0 mg/dL.

We examined the clinical courses of corrected serum Ca, serum Cr, and estimated glomerular filtration rate (eGFR) after treatment. We defined Ca-1, Cr-1, and eGFR-1 as the values at the time of admission, Ca-2, Cr-2, and eGFR-2 as the values when serum Ca level returned to a normal level for the first time after treatment, and Ca-3, Cr-3 as the lowest and eGFR-3 highest values after treatment. Treatment #1 was a discontinuation of ELD. Treatment #2 included treatment #1 and rehydration with isotonic saline. Treatment #3 included treatment #2 and a calcitonin injection. Allocation of treatment was determined by the attending physicians based on the level of hypercalcemia and AKI. To further determine the impact of concomitant medication on the degree of hypercalcemia and AKI, we compared eGFR and serum levels of Ca and Cr on admission with or without treatment with the following drugs: RAASIs, NSAIDs, loop diuretics, magnesium oxide, and bisphosphonates.

### Statistical analysis

Parametric variables with a normal distribution were expressed as mean ± SD. Categorical data were expressed numerically or as percentages. Parametric variables between 2 groups were compared using an unpaired *t*-test and those among 3 groups were compared by one-way analysis of variance. Correlation coefficients were calculated to determine whether there was a correlation between 2 parametric variables. Dunnett’s test was used to determine whether there was a difference compared with controls. All statistical analyses were performed with EZR software (Saitama Medical Center, Jichi Medical University, Saitama, Japan), which is a graphical user interface for R (The R Foundation for Statistical Computing, Vienna, Austria), and a modified version of R commander designed to add statistical functions frequently used in biostatistics [10]. A two-tailed value of *p* < .05 was considered statistically significant.

## Results

### All patients with hypercalcemia-induced AKI

To determine the prevalence and cause of AKI in our hospital, we first reviewed the medical charts and found that a total of 69 patients presented with hypercalcemia-induced AKI between April 2013 and February 2018 to our facility. The etiology of the hypercalcemia cases is shown in Table 1. Among 69 cases of hypercalcemia, 43 cases were caused by VDRAs: alfacalcidol in 9 cases; calcitriol in 2 cases; ELD in 32 cases.

**Table 1.** Causes of hypercalcemia-induced AKI in the examined 69 patients.

Causes	Number
Eldecalcitol	32
Alfacalcidol	9
Malignancy-associated hypercalcemia	9
Calcium-alkali syndrome	5
Primary hyperparathyroidism	3
Sarcoidosis	3
Immobilization	3
Calcitriol	2
Familial hypocalciuric hypercalcemia	2
Vitamin A intoxication	1
Total	69

AKI: acute kidney injury.

### Participant characteristics

The detailed demographic and clinical information of all the 32 patients are shown in Table 2. To determine the clinical characteristics at baseline in patients with ELD-induced AKI, we investigated the 32 patients who developed AKI secondary to ELD-induced hypercalcemia. Table 3 summarizes the patients’ characteristics on admission. Mean age was 82 ± 5 years, 97% of participants were female, mean body mass index was 21 ± 4 kg/m^2^, and 13% had a history of diabetes. Mean corrected serum Ca level was 12.2 ± 1.5 mg/dL, serum Cr level was 2.5 ± 2.2 mg/dL, eGFR was 23.9 ± 14.4 mL/min/1.73 m^2^, intact parathyroid hormone was 22.5 ± 18.8 pg/mL, urinary Ca to Cr ratio was 0.25 ± 0.17 and hydrogen carbonate was 29.1 ± 4.3 mmol/L. All of the patients were treated with 0.75 µg/day of ELD and none was treated with 0.50 µg/day of ELD.

**Table 2.** Demographic and clinical information of 32 patients.

Case	AKI Stage	Age (years)	Sex	Dose of ELD (µg/day)	Serum Ca (mg/dL)	Ca^2+^ (mmol/L)	Serum Cr (mg/dL)	eGFR (mL/min/1.73 m^2^)	Serum Pi (mg/dL)	Intact PTH, (pg/mL)	U-Ca/U-Cr ratio	pH	HCO_3_^−^ (mmol/L)	Treatment	Outcome
1	1	70	F	0.75	10.4	N/A	0.84	51.5	3.6	N/A	0.08	N/A	N/A	#1	Resolution
2	1	73	F	0.75	10.2	1.26	1.4	29	3.1	N/A	N/A	7.40	26.5	#1	Resolution
3	1	73	F	0.75	11.8	N/A	1.49	27.1	3	48	N/A	N/A	N/A	#1	Resolution
4	1	75	F	0.75	11.5	N/A	1.15	35.8	2.8	N/A	N/A	N/A	N/A	#1	Resolution
5	1	78	F	0.75	11.9	1.46	1.44	27.6	2.9	12.3	N/A	7.45	32.8	#1	Resolution
6	1	79	F	0.75	11.9	N/A	0.97	42.5	4.2	N/A	0.49	N/A	N/A	#1	Resolution
7	1	81	M	0.75	11.4	1.45	2.56	19.7	3.6	16	0.2	7.40	27	#1	Resolution
8	1	82	F	0.75	10.4	N/A	0.65	64.8	3.9	N/A	N/A	N/A	N/A	#1	Resolution
9	1	84	F	0.75	11.9	1.46	2.92	12.4	4.1	25	0.18	7.35	25.1	#3	Resolution
10	1	87	F	0.75	11.1	N/A	1.5	25.6	3.6	9.3	0.49	N/A	N/A	#1	Resolution
11	1	88	F	0.75	10.5	N/A	1.56	24.5	3.6	N/A	N/A	7.32	29.9	#1	Resolution
12	1	88	F	0.75	12.4	N/A	0.9	44.5	3.2	12.7	N/A	N/A	N/A	#1	Resolution
13	2	77	F	0.75	10.1	N/A	0.93	44.8	3.3	N/A	0.05	N/A	N/A	#1	Resolution
14	2	78	F	0.75	12.5	1.3	1.36	29.3	2.8	17.6	0.42	7.44	27.8	#1	Resolution
15	2	79	F	0.75	11.4	1.43	1.88	20.6	3.9	14.8	0.13	7.39	25.3	#2	Resolution
16	2	81	F	0.75	16.1	1.94	2.19	17.3	2.3	13.2	0.16	7.48	33.3	#3	Resolution
17	2	81	F	0.75	13.1	1.65	4.01	8.9	4.5	13.1	N/A	7.35	20.6	#2	Resolution
18	2	83	F	0.75	13.6	1.69	3.07	11.8	3.8	26.1	0.39	7.42	28.2	#3	Resolution
19	2	84	F	0.75	12.1	1.43	2.11	17.8	4.1	N/A	N/A	7.37	35.5	#1	Resolution
20	2	85	F	0.75	11.6	1.38	1.12	36.1	2.8	15	N/A	7.38	39.1	#1	Resolution
21	2	85	F	0.75	12.1	N/A	2.56	14.3	3.3	N/A	N/A	N/A	N/A	#1	Resolution
22	2	85	F	0.75	11.7	N/A	1.83	20.8	3.5	8.8	N/A	N/A	N/A	#1	Resolution
23	2	89	F	0.75	13.1	1.64	1.36	28.2	3.3	14	0.57	7.40	32.3	#3	Resolution
24	3	80	F	0.75	15	N/A	3.5	10.4	5.6	89.5	0.04	N/A	N/A	#3	Resolution
25	3	80	F	0.75	12.2	1.51	13.11	2.4	5.1	N/A	0.24	7.40	26.1	#3	Resolution
26	3	80	F	0.75	11.8	N/A	3.09	11.9	3.9	14.2	0.27	N/A	N/A	#2	Resolution
27	3	81	F	0.75	13.2	1.66	2.21	17.1	3.4	17.2	0.34	7.40	26.9	#2	Resolution
28	3	82	F	0.75	11.2	N/A	1.33	29.6	3.9	N/A	N/A	N/A	N/A	#1	Resolution
29	3	82	F	0.75	10.6	1.37	3.36	10.8	4	N/A	N/A	7.35	26.6	#1	Resolution
30	3	88	F	0.75	14.1	1.72	4.65	7.4	4.4	32.2	N/A	7.38	27.9	#3	Resolution
31	3	88	F	0.75	13.4	1.39	2.77	13	4	N/A	N/A	7.39	28.8	#2	Resolution
32	3	90	F	0.75	15.6	1.89	4.72	7.2	4.2	27.6	0.25	7.47	32.9	#3	Resolution

Patients were treated with one of the following treatment options: Treatment #1, Discontinuation of eldecalcitol; Treatment #2; Treatment #1 + hydration with isotonic saline; Treatment #3, Treatment #2 + calcitonin injection. Stage of AKI was based on the Kidney Disease Improving Global Outcomes Guideline 2012.

AKI: acute kidney injury; Ca: calcium; Ca^2+^: ionized calcium; Cr: creatinine; eGFR: estimated glomerular filtration rate; ELD: eldecalcitol; F: female; HCO_3_^−^: hydrogen carbonate; M: male; N/A: not available; Pi: Inorganic phosphorus; PTH: parathyroid hormone; U: urinary.

**Table 3.** Summary of the clinical backgrounds on admission of the 32 patients with hypercalcemia induced by ELD.

Demographic data		Complaints and symptoms	
Age, years	82 ± 5	AKI	32 (100)
Sex,female	31 (97)	Xerostomia and polyuria	9 (26)
Diabetes mellitus	4 (13)	Disturbance of consciousness	4 (13)
Height, cm	146 ± 7	Nausea, Vomiting	2 (6)
Body weight, kg	44 ± 9	The number of attending hospitals	
Body mass index, kg/m^2^	21 ± 4	Single hospital	9 (28)
Systolic blood pressure, mmHg	137 ± 23	Multiple hospitals	23 (72)
Diastolic blood pressure, mmHg	73 ± 15	Prescribers of ELD	
Heart rate, bpm	81 ± 12	Orthopedic surgeons	20 (63)
Blood and Urinary tests		General practitioners	11 (34)
eGFR, mL/min/1.73m^2^	23.9 ± 14.4	Rheumatologists	1 (3)
Serum Cr, mg/dL	2.5 ± 2.2	Concomitant medications	
Corrected serum Ca, mg/dL	12.2 ± 1.5	NSAIDs	11 (34)
Serum phosphate, mg/dL	3.7 ± 0.7	Adrenocortical steroid	4 (13)
Serum alkaline phosphatase, U/L	228 ± 109	Bisphosphonate	9 (28)
Intact PTH, pg/mL	22.5 ± 18.8	Raloxifene hydrochloride	4 (13)
U-Ca/U-Cr ratio	0.25 ± 0.17	Magnesium oxide	11 (34)
pH	7.40 ± 0.04	Calcium channel blocker	21 (66)
HCO_3_^−^, mmol/L	29.1 ± 4.3	RAASIs	16 (50)
Dose of ELD		Loop diuretics	8 (25)
0.75 µg/day	32 (100)	Thiazide diuretics	2 (6)
0.5 µg/day	0 (0)	Calcium supplements	2 (6)

Data are expressed as mean  ±  SD or number (percentage).

AKI: acute kidney injury; Ca: calcium; Cr: creatinine; eGFR: estimated glomerular filtration rate; ELD: eldecalcitol; HCO_3_^−^: hydrogen carbonate; NSAIDs: non-steroidal anti-inflammatory drugs; PTH: parathyroid hormone; RAASIs: renin-angiotensin-aldosterone system inhibitors; U: urinary.

The prescribers of ELD included orthopedic surgeons (63%), general practitioners (34%), and rheumatologists (3%). Seventy-two percent of the patients had been treated by multiple doctors at different medical facilities. Thirty-four percent of the patients used NSAIDs, 13% used adrenocortical steroids, 28% used bisphosphonates, 34% used magnesium oxide, 66% used calcium channel blockers, 50% used RAASIs, 25% used loop diuretics, 6% used thiazide diuretics, and 6% used calcium supplements.

### Comparison of the background data on admission between patients either treated or not treated with ELD

We compared the clinical backgrounds of patients either treated or not treated with ELD in our study. As shown in Table 4, the mean age and the proportion of females were significantly (*p* < .05) higher in the ELD group than those in non-ELD group.

**Table 4.** Comparison of the background data on admission between patients either treated or not treated with ELD.

Variables	ELD (*n* = 32)	Non-ELD (*n* = 37)	*p* value
Age, year	82 ± 5	73 ± 13	*p* < .05
Sex, female	31 (97)	19 (51)	*p* < .05
Serum Cr, mg/dL	2.5 ± 2.2	2.3 ± 1.4	.341
Corrected serum Ca, mg/dL	12.2 ± 1.5	12.2 ± 1.7	.474
Concomitant medication			
NSAIDs	11 (34)	8 (30)	.187
RAASIs	16 (50)	10 (27)	.132
Loop diuretics	8 (25)	7 (19)	.572
Bisphosphonates	9 (28)	5 (14)	.148
Magnesium oxide	11 (34)	8 (22)	.286

Data are expressed as mean  ±  SD for parametric variables or number (%) for nominal data. A *p* value less than .05 was considered statistically significant. Un-paired *t*-test was used for the comparison of parametric data and chi-square test was used for nominal data.

Ca: calcium; Cr: creatinine; ELD: eldecalcitol; NSAIDs: non-steroidal anti-inflammatory drugs; RAAS: renin-angiotensin-aldosterone system.

### Clinical course of ELD-induced AKI

**Figure 1.**
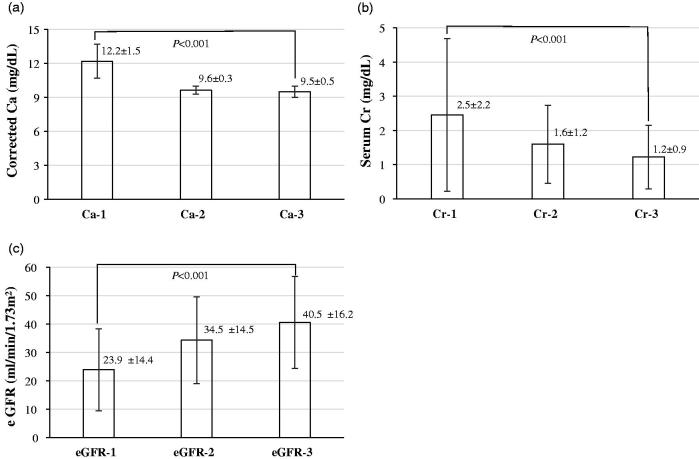
Clinical courses of (a) corrected serum Ca, (b) serum Cr, and (c) eGFR. Ca-1, Cr-1, and eGFR-1 were values on admission. Ca-2, Cr-2, and eGFR-2 were values when serum Ca level was normalized for the first time after treatment. Ca-3 and Cr-3 were the lowest values, eGFR-3 was the highest value after treatment. Ca: calcium; Cr: creatinine; eGFR: estimated glomerular filtration rate. Data are expressed as mean ± SD. Data were compared by Dunnett test by setting data on admission (Ca-1, Cr-1, or eGFR-1) as controls. A *p* value less than .05 was considered statistically significant.

The clinical courses of eGFR and corrected serum Ca and Cr after treatment in patients with ELD-induced AKI are shown in Figure 1. All three parameters were significantly improved by treatment (*p* < .05). The clinical background of the patients stratified by selected treatment is shown in Table 5. All patients discontinued administration of ELD. Additionally, 16% of the patients received hydration therapy (treatment #2), 25% of the patients received combination treatment with hydration and calcitonin (treatment #3), and the remaining patients simply discontinued ELD (treatment #1). There were significant differences in corrected serum Ca (*p* < .05) and serum Cr (*p* < .05) on admission, and reduction rate of serum Ca (*p* < .05) and serum Cr (*p* < .05) from the value on admission. In addition, serum Ca levels in patients receiving treatment #2 and #3 returned to normal levels significantly faster than those who received treatment #1.

**Table 5.** Clinical background of the patients stratified by treatment.

Treatment	#1	#2	#3	*p* value
*N* = 32, (%)	19 (59)	5 (16)	8 (25)	
Corrected serum Ca, mg/dL	11.6 ± 0.7	12.6 ± 0.9	14.3 ± 1.6	*p* < .05
Serum Cr, mg/dL	1.7 ± 0.7	2.8 ± 0.8	3.2 ± 1.3	*p* < .05
Reduction rate of serum Ca level from the value on admission, %	18 ± 5.1	26 ± 3.9	33 ± 10.7	*p* < .05
Reduction rate of serum Cr level from the value on admission, %	32 ± 16.8	60 ± 13.3	59 ± 18.1	*p* < .05
Time required for normalization of serum Ca level, day	37	15	14	*p* = .154

Data are expressed as mean ± SD, percentage, or number (percentage). One-way ANOVA was used to determine whether there was a difference in the values. A *p* value less than .05 was considered statistically significant. Patients were treated with one of the following treatment options: Treatment #1, Discontinuation of eldecalcitol; Treatment #2; Treatment #1 + hydration with isotonic saline; Treatment #3, Treatment #2 + calcitonin injection.

Ca: calcium; Cr: creatinine.

### The impact of hypercalcemia on the magnitude of serum Cr change

**Figure 2.**
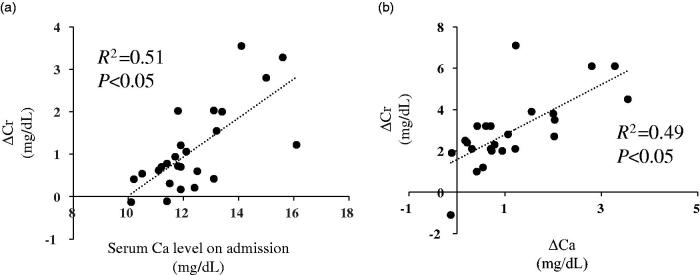
Correlation between ΔCr change and baseline serum Ca level or Δ serum Ca level. Correlation between Δ serum Cr and (a) serum Ca level on admission and (b) Δ serum Ca level. To determine the correlation between two continuous variables, Pearson’s correlation coefficient was determined. Δ serum Cr (mg/dL) = (serum Cr level on admission) – (serum Cr level after treatment). Δ serum Ca (mg/dL) = (serum Ca level on admission) – (serum Ca level after treatment). A *p* value less than .05 was considered statistically significant. Ca: calcium; Cr: creatinine.

To determine the impact of hypercalcemia on AKI, we conducted a simple correlation analysis. Δ serum Cr level was significantly and positively correlated with serum Cr level on admission and Δ serum Ca level (Figure 2).

### Comparison of eGFR and serum levels of Ca and Cr on admission stratified by concomitant medication usage

**Figure 3.**
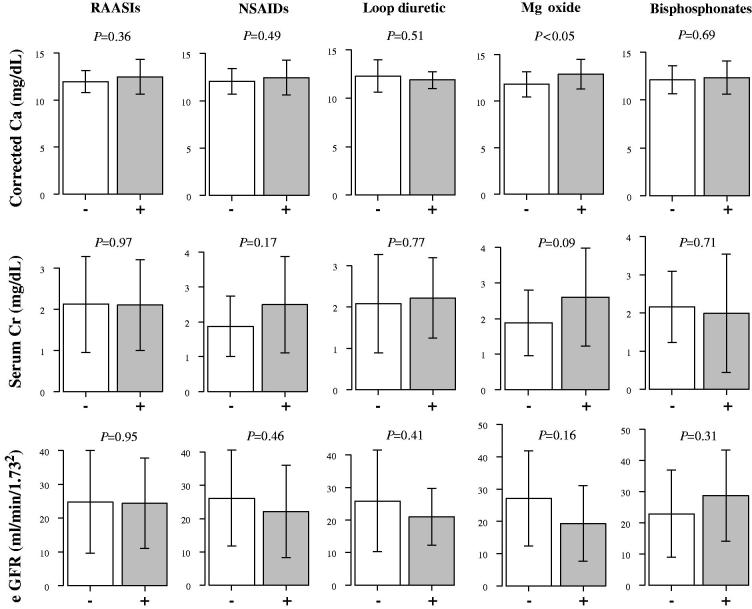
Comparison of eGFR and serum Ca and Cr on admission stratified by concomitant medication use. Ca: calcium; Cr: creatinine; eGFR: estimated glomerular filtration rate; Mg: magnesium; NSAIDs: non-steroidal anti-inflammatory drugs; RAASIs: renin-angiotensin-aldosterone system inhibitors. Data are expressed as mean ± SD. Data between two groups were compared using an unpaired *t*-test. A *p* value less than .05 was considered statistically significant.

To determine the impact of concomitant medication usage on eGFR and the serum levels of Ca and Cr, we stratified those data by medications taken at the time of admission (Figure 3). Corrected serum Ca level on admission was significantly high when magnesium oxide was used (*p* < .05). In contrast, the corrected serum Ca level on admission tended to be lower when loop diuretics were used. Serum Cr levels on admission tended to be higher when NSAIDs and magnesium oxide were used. However, there were no statistically significant differences in the other analyses, likely because of insufficient study power.

## Discussion

In this study, we showed that 46.4% of AKI cases with hypercalcemia at our facility were associated with ELD. After the diagnosis of ELD-induced AKI, all patients discontinued administration of ELD and their serum Ca levels returned to normal, and eGFR increased significantly. A correlation study showed that the magnitude of AKI was higher when patients showed a higher serum Ca level on admission. Regarding the characteristics of the 32 patients with ELD-induced AKI, there was a trend for the proportion of females to be higher (97%) and the patients to be older (mean age was 82 ± 5 years) compared with patients in previous studies [5,7]. Corrected serum Ca level on admission in patients treated with magnesium oxide was significantly higher than those without. Although not statistically significant, some concomitant medications that affect renal hemodynamics or Ca metabolism tended to increase the risk for ELD-induced AKI.

High serum levels of 25-hydroxyvitamin D and 1,25-dihydroxyvitamin D can cause hypercalcemia by increasing Ca absorption from the gastrointestinal tract and increasing bone resorption [11]. Intestinal transport of Ca is primarily regulated by 1,25-dihydroxyvitamin D. In the clinical setting, hypercalcemia occurs in patients who ingest high doses of vitamin D or vitamin D analogs for disorders such as osteoporosis [12]. Since ELD is a form of VDRAs, ELD can induce hypercalcemia via the same mechanism described above. ELD has a higher affinity for vitamin D-binding protein than calcitriol [13] and increases the affinity to the serum carrier proteins and prolongs its half-life to 53 h [14]. Therefore, ELD has a stronger effect on Ca absorption from the intestinal tract than the other conventional VDRAs such as alfacalcidol or calcitriol, which is indirectly supported by our observation that ELD was the leading cause of hypercalcemia among the cases of VDRA-related hypercalcemia.

As for the potential mechanisms of hypercalcemia-induced AKI, there are at least three explanations. First, hypercalcemia causes spasm of the renal afferent arteries, thereby decreasing glomerular filtration rate. Second, tubulointerstitial injury or fibrosis is induced by chronic Ca deposition in the kidneys. Finally, dehydration with a failure in urinary concentration is caused by decreased reactivity of antidiuretic hormone in the renal collecting duct through a Ca-sensing receptor [15]. AKI associated with ELD-induced hypercalcemia is explained by a combination of these mechanisms.

A variety of risk factors for hypercalcemia due to ELD likely exist. In Japan, 40% of women over 65 years old are CKD (estimated GFR ≤ 60 mL/min/m^2^) patients [16]. Indeed, another study showed that 19.2%–26.5% of women aged 40 years or older are osteoporotic at the lumbar spine and femoral neck [17]. This means that the clinical background of postmenopausal women with osteoporosis is similar to that of CKD patients. Since CKD involves a decreased capacity for excreting Ca in the urine, CKD increases the risk for hypercalcemia when these patients are treated with calcium or VDRAs. Previous research has shown that 59% of patients with hypercalcemia due to ELD had CKD, 93% were female, and 83% were over 70 years old [7]. Our study also showed that patients with hypercalcemia were older (82 ± 5 years) and mostly female (97%). Actually, the mean age and the proportion of females were significantly higher in the ELD group than in the non-ELD group in our study. The reason for the higher age in the ELD group was that ELD was only used for osteoporotic patients, while the non-ELD group included patients with hypercalcemia induced by a variety of causes that can affect younger patients as well. These results suggest that patients with osteoporosis frequently complicate CKD and are potentially prone to develop hypercalcemia when they are treated with VDRAs because their capacity to increase urinary Ca excretion was decreased.

Another potential risk factor for ELD-induced AKI is concomitant medications that patients may be taking that affect renal hemodynamics and renal calcium metabolism. NSAIDs cause spasm of renal afferent arteriole [18], and RAASIs have a vasodilatory effect on the efferent arteriole [19], which decreases glomerular filtration rate and leads to decreased urinary Ca excretion and an increased serum Ca level. Magnesium oxide is an absorbent alkaline and is associated with milk-alkali syndrome. Milk-alkali syndrome is characterized by the triad of hypercalcemia, metabolic alkalosis, and diminished kidney function [20]. Metabolic alkalosis, initiated by alkali ingestion, increases the affinity of Ca sensing receptor for Ca, thereby enhancing the inhibition of sodium and Ca reabsorption [21]. Even in our study, patients tended to exhibit metabolic alkalosis (hydrogen carbonate was 29.1 ± 4.3 mmol/L, Table 3). In such a mechanism, corrected serum Ca levels on admission might have been higher when magnesium oxide was used in our study.

Thiazide diuretics reduce urinary Ca excretion, whereas loop diuretics increase it [22]. The former can increase serum Ca level, while the latter can reduce it. In our 8 cases who used a loop diuretic, the serum corrected Ca level on admission tended to be lower. Additionally, loop diuretics can increase the risk for AKI by decreasing extracellular fluid volume. Importantly, older patients often have hypertension, orthopedic complications, and constipation, and they are often treated with RAASIs, diuretics, NSAIDs, and magnesium oxide. Although there was no significant difference in eGFR or serum levels of Ca and Cr between patients who did or did not use these drugs, prescribers of ELD should be careful about patients’ concomitant medications, especially when patients with osteoporosis are older, female, have CKD, or are users of NSAIDs, RAASIs, diuretics, and magnesium oxide. Our hypothesis should be further confirmed with case-control studies or prospective observational studies.

Based on our survey, prescribers of ELD might not have been aware of other concomitant medications being taken by patients when they prescribed ELD because 72% of the patients had been treated by multiple doctors at different medical facilities. Because some drugs such as RAASIs, NSAIDs, diuretics, and magnesium oxide increase the risk for AKI and hypercalcemia, it is of great importance for ELD prescribers to know what medications have been prescribed to their patients at other medical facilities. Close follow-up and periodic monitoring of serum Cr and Ca levels after ELD prescription are highly recommended to avoid hypercalcemia and resulting AKI. Besides, we should be aware of the concomitant use of drugs that heighten the risk for hypercalcemia. Such efforts would be expected to reduce the risk for ELD-induced hypercalcemia and AKI.

Since a previous report showed the superiority of 0.75 µg/day of ELD over 1 µg/day of ALF [5], ELD prescribers tend to choose 0.75 µg/day more frequently than 0.5 µg/day. All of the patients in our study received 0.75 µg/day of ELD instead of 0.5 µg/day. Given that 0.75 µg/day of ELD has a stronger titer than 0.5 µg/day, it is not surprising that 0.75 µg/day of ELD causes hypercalcemia more frequently than 0.5 µg/day. Furthermore, 97% of the prescribers who referred patients with ELD-induced AKI to our facility did not provide any information on serum Ca level, indicating that they did not consider hypercalcemia associated with ELD in the differential diagnosis as a cause of AKI. Our study strongly suggests that periodic monitoring of serum Ca level in patients receiving 0.75 µg/day of ELD should be performed to detect mild increases in hypercalcemia and early-stage AKI. This strategy would likely decrease the incidence of ELD-induced hypercalcemia and AKI and decrease the need for intensive treatment and hospitalization.

Several limitations of our study need to be considered. First, this was a single-center, retrospective study with a small sample size. Second, we cannot deny that other drugs such as NSAIDs, RAAS inhibitors, and thiazide diuretics (as opposed to the ELD) already prescribed at the time of admission may have contributed to the hypercalcemia-induced AKI in some of the patients recruited into this study. Third, in the analysis of eGFR and serum levels of Ca and Cr, on admission stratified by concomitant medications, we were unable to show significant differences with the use of many of the drugs because of the lack of study power, except for magnesium oxide. However, based on our personal experience, patients treated with ELD are likely to develop hypercalcemia when they are female, older, have a lower body weight, have CKD, or are treated with NSAIDs, RAASIs, thiazide diuretics. Fourth, our study did not have appropriate controls, namely patients who were treated with ELD but did not develop hypercalcemia. Thus, we could not evaluate the true risk factors for hypercalcemia and AKI associated with ELD. Finally, unfortunately, we had no data on the duration of ELD treatment at the time of admission. This was because most of the patients with ELD-induced AKI in our study were referred to our facility by clinicians working at other clinics and hospitals. Hence, further studies, including case-control studies or larger observational studies with detailed clinical background information, are necessary to determine the pathophysiology of ELD-associated hypercalcemia and resulting AKI.

## Conclusion

The present study showed that ELD is the leading cause of hypercalcemia-induced AKI at our hospital. Moreover, patients treated with ELD tended to be those who were older and female, those with preexisting CKD, and those treated with drugs that affect renal hemodynamics and Ca metabolism, and they tended to be at increased risk for hypercalcemia and resulting AKI. Prescribers of ELD should be aware that 0.75 μg/day of ELD for the treatment of osteoporosis potentially increases the risk for hypercalcemia and related AKI and should regularly monitor serum calcium levels and kidney function to prevent ELD-induced hypercalcemia and AKI. Further studies such as case-control studies or larger observational studies are necessary to further determine the risk factors for ELD-induced hypercalcemia and AKI.
